# A reduced activity model: a relevant tool for the study of ageing muscle

**DOI:** 10.1007/s10522-015-9613-9

**Published:** 2015-10-27

**Authors:** Oliver Perkin, Polly McGuigan, Dylan Thompson, Keith Stokes

**Affiliations:** Department for Health, University of Bath, Bath, BA2 7AY UK

**Keywords:** Step-reduction, Muscle, Strength, Atrophy, Inactivity, Ageing

## Abstract

Skeletal muscle mass is in a constant state of turnover, and atrophy is the result of a shift in the balance of muscle protein synthesis and breakdown resulting in net muscle protein loss. Total disuse of skeletal muscle quickly leads to muscle atrophy and loss of strength, and this has been repeatedly demonstrated in studies employing bed rest and lower limb immobilisation methodologies in young healthy participants. Fewer studies have focused on older participants (>65 years of age), but those that have provide evidence that advancing age brings increased vulnerability to rapid and marked loss of muscle size and strength during period of total muscle unloading. Increased systemic inflammation and reduced protein synthetic responses to protein feeding and muscle contraction might influence the severity of muscle protein loss during periods of total unloading compared with younger individuals. Less extreme reductions in muscle loading (e.g., 2 weeks of reducing daily ambulation to <1500 steps/day) have also been shown to result in decreases in muscle mass. This step-reduction model may be more relevant than total bed rest or limb immobilisation for examining real-world scenarios that present a physiological challenge to the maintenance of skeletal muscle mass in older individuals.

## Introduction

The importance of maintaining adequate skeletal muscle mass and strength throughout the lifespan is well recognised. Ageing is associated with decreasing muscle mass (sarcopenia), and evidence is unequivocal that periods of unloading of skeletal muscle also cause reductions in muscle cross-sectional area (mCSA), volume, mass, and strength in both young and older populations (Bodine 2013; Coker et al. 2015; Deschenes et al. 2008; Hvid et al. 2010; Kortebein et al. 2007; Rejc et al. 2015; Suetta et al. 2009; Wall et al. 2013). The annual rate of muscle mass loss after the age of 50 years is estimated to be around 0.5–1 % (Mitchell et al. 2012), although this figure is subject to large variation between individuals due to a combination of genetic and lifestyle factors, and between muscle groups within individuals, with lower limb muscles typically exhibiting greater rate of atrophy with age than upper limb muscles (Degens and Korhonen 2012). Whilst a clear relationship exists between mCSA and force generating capacity in healthy, young individuals, it is apparent that the rate of strength loss with age is accelerated compared to mCSA loss (Seene and Kaasik 2012). The loss of muscle strength with age is commonly termed dynapenia, and is regarded as a primary cause of the loss of functional independence in older adults. Furthermore, decline in muscle mass in any population is generally allied with negative changes to body composition, i.e. accrual of fat mass (both in absolute terms and relative to lean tissue), and the associated deterioration of metabolic health and elevation in markers of systemic inflammation derived from adipose tissue (Cesari et al. 2005; Patel et al. 2011). Both ageing and physical inactivity are also independently associated with increased systemic inflammation and oxidative stress, with circulating inflammatory cytokines [tumour necrosis factor-α (TNF-α), Interleukin-6 (IL-6), C-reactive protein (CRP)] implicated in further potentiation of muscle wasting (Cesari et al. 2005; Schaap et al. 2006; Visser et al. 2002).

Maintenance of muscle mass is the result of continual steady turnover of muscle protein. Research exploring mechanisms for skeletal muscle loss has been centred on the basic concept that the balance of concurrent muscle protein synthesis (MPS) and muscle protein breakdown (MPB) shifts towards a state of net muscle protein loss (Gibson et al. 1987), with comprehensive reviews of the mechanisms available (Burd et al. 2012; Hackney and Ploutz-Snyder 2012; Phillips et al. 2009; Rennie et al. 2004). To clarify the minutiae of this negative shift in muscle protein turnover brought about by muscle disuse in humans, previous studies have used extreme interventions to unload participants’ muscles such as prolonged periods of bed rest or unweighting of a limb, producing stark physiological changes that have been readily quantifiable (Coker and Wolfe 2012; Rittweger et al. 2005; Trappe et al. 2008; Trappe et al. 2004). These complete models of unloading have provided a wealth of knowledge on the physiological processes at play during muscle wasting in various populations and muscle groups. However, total unloading in real world scenarios are likely to be accompanied by severe challenges to physiological homeostasis such as disease and injury, with these complete disuse models often providing a surrogate for zero gravity space flight which bears little applicability to older individuals. Furthermore, in older individuals severe health challenges resulting in bed-rest are commonly associated with protein under-nutrition, itself exacerbating muscle loss, which is ethically challenging to include in clinical studies (Sullivan et al. 1999; Tieland et al. 2012). Common scenarios faced by healthy older individuals in everyday life that lead to reduced physical activity may include sustained inclement weather, suffering a minor injury or illness, or undergoing elective surgery which do not result in extended total unloading (Cohen-Mansfield et al. 2003; Grossman and Stewart 2003). The aim of this review is to highlight a potentially less severe model of reduced activity (limiting daily ambulation) to explore the role of more common events in the progression of sarcopenia in an ageing population.

## Total unloading models and exaggerated disuse induced muscle loss with ageing

Traditionally research exploring the effect of unloading of muscles using human participants has tended to implement models of entire lower body muscle disuse through bed-rest, or unilateral limb disuse via immobilisation by casting, bracing, or suspension. Total unloading interventions, in particular bed rest with a 6° head-down tilt (HD), have commonly been used to mimic the effects of zero gravity space flight on muscle and bone turnover. These studies have consistently demonstrated that total unloading of muscle induces substantial declines in muscle mass and strength. For example, young, healthy male participants undertaking HD bed rest for 90-days experienced a 26 ± 3 % decline in calf mCSA measured using peripheral quantitative computed tomography (pQCT) (Rittweger et al. 2005). In fact, even participants performing 28 maximal concentric and eccentric supine squats and calf press every third day for the duration of bed rest still lost 17 ± 3 % of calf mCSA. Using magnetic resonance imaging (MRI) Trappe et al. (2004) reported a 17 % loss of quadriceps muscle volume from baseline in a group of 6 healthy males following 84-days of HD bed rest when provided no protective intervention. Of note, maximum voluntary contraction (MVC) during isometric squats was reduced by 43 % in this group, and peak power by 47 %. Whilst these are both examples of long term and complete unloading, even relatively short duration disuse has been shown to induce marked reductions in muscle size. A study by Suetta et al. (2009) observed quadriceps muscle volume decreases of 8.9 % in young males subjected to 14-days of limb immobilisation through casting, with the knee set at 30° flexion to prevent any load bearing. These findings are comparable with the 5 ± 1 % decline in quadriceps mCSA reported by Glover et al. (2008) using a similar 2 week immobilisation intervention, which also induced a 25 ± 3 % drop in peak isometric torque.

A study by Miokovic et al. (2012) examining muscle volume from MRI of the whole lower limb pre-, mid-way through, and post- 60-day HD bed rest highlighted the non-uniformity in mCSA loss between individual muscles. All muscles of the lower limb were measured in this study, with the most pronounced atrophy occurring in the gastrocnemius medialis and soleus, and then vasti muscles of the thigh, followed by tibialis anterior, the hamstrings, and adductor magnus. Furthermore, atrophy was not uniform across the muscle length in 12 of the 19 muscles measured, and the muscle sub-region of greatest atrophy did not necessarily correlate with the point of greatest mCSA. Uniform atrophy only occurred in the soleus, adductor brevis, gracilis, pectineus, and extensor digitorum longus muscles. The authors suggest that reasons for differential atrophy between muscles and also across muscle lengths are both likely to be related to the specific use of the individual muscles and sub-regions of those muscles in everyday life, with the muscles and sub-regions used most often suffering the greatest disuse induced atrophy. Anatomical differences between muscles also likely plays a part in explaining inter-muscle differences in atrophy rate, however it was noted that information regarding functional or anatomical compartmentalisation for a number of lower limb muscles was not present in the literature for humans. This information may be important when examining loss of muscle force generating capability across a range of joint angles after a period of unloading inducing muscle loss.

Older individuals show an amplified susceptibility to lose muscle mass through total lower limb disuse compared to younger individuals (Degens and Korhonen 2012; Tanner et al. 2015). As such, due to the ethical implications of intentionally inducing muscle wasting the participants of previous total unloading studies have tended to be healthy young individuals, especially in studies implementing bed rest. Using DEXA, 10-days of bed rest has been demonstrated to induce reductions in leg lean tissue mass of 7 % in participants aged 68 ± 5 years (Kortebein et al. 2006), and a loss of two kilograms of whole body lean mass, of which one kilogram was from the lower extremities in participants aged 67 ± 2 years (Deutz et al. 2013). This rapid loss of muscle mass in older individuals is consistent with findings from a 7 day bed rest study involving six 60–73 year olds, observing losses of 3.0 % of total lean mass, and 4.1 % of lean leg mass (Drummond et al. 2012). Despite the duration of bed-rest in these studies with older participants being less than half of that used by Paddon-Jones et al. (2004), in absolute terms these losses are twice that observed in participants aged 38 ± 6 years following 28-days of bed rest.

Loss of muscle function also seems to be exaggerated in older individuals compared to younger individuals in response to complete unloading (Trappe 2009). Both isometric muscle strength relative to muscle volume and the rate of force development decreased significantly more in the older than younger men following 2 weeks of unilateral leg casting (Hvid et al. 2010). Surprisingly, older individuals lost relatively less individual muscle fibre size, and only showed significant cross sectional area (CSA) loss of type IIa fibres whereas younger individuals lost CSA in all three fibre types. This aligns with the findings of Nilwik et al. (2013) that reduced type II muscle fibre size is the main cause of skeletal muscle mass loss with ageing. Type II fibres are generally correlated with explosive movements, but Hvid et al. (2010) did not identify any significant associations between loss of type II fibre area and rate of force development capacity after the immobilisation period in either age group. This likely implicates neural changes in the loss of muscle function during limb unloading, especially in the older group. Previously reported data from the same investigation demonstrated that older individuals displayed reduced neuromuscular activation capabilities during maximal voluntary contraction, which the younger group did not (Suetta et al. 2009).

Recovery of lost muscle mass due to unloading is likely to be more challenging in older individuals compared to younger individuals. Kumar et al. (2012) demonstrated that older men required an increased volume of resistance exercise to match the MPS rates achieved by younger men. This supports findings in older women, whereby 12 weeks of resistance training, three times per week, induced 6.2 % increases in quadriceps muscle volume in younger women compared to only 2.5 % increases in older women (Greig et al. 2011). However, marked gains in both muscle size and strength can be achieved in older individuals undertaking resistance exercise regimes. For instance, in a recent study by Leenders et al. (2013) quadriceps mCSA increased by 8 % in women and 7 % in men after 12 weeks of resistance exercise, with no increase following a further 12 weeks of training. At 12 weeks, strength measured by leg extension 1-RM increased by 22 % in females and 23 % in males, and by 24 weeks had further increased by 17 % in females and 16 % in males, despite no further increase in quadriceps muscle size. Also, older females completing a 12-week whole body progressive resistance training programme using machine weights increased rectus femoris muscle volume by 26 % measured using ultrasound (Correa et al. 2013).

**Fig. 1:**
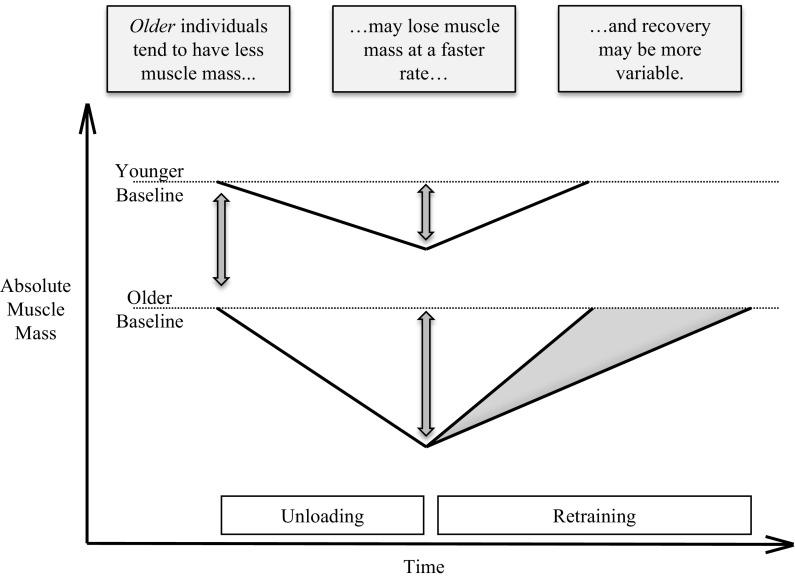
Schematic of the differences in muscle mass changes in older compared to younger individuals in response to match unloading and retraining protocols

Resistance exercise regimes undertaken specifically to recover muscle size and strength after disuse induced atrophy in an older population have received notably less attention in the literature. A study employing 4 weeks of retraining after 2 weeks of unilateral limb immobilisation in older and younger individuals suggests that recovery of muscle size and some aspects of strength may be impaired in older individuals (Hvid et al. 2010; Suetta et al. 2009). Specifically, Suetta et al. (2009) observed that the retraining period did not restore older individuals’ quadriceps muscle volume to pre-immobilisation values, whilst the younger group fully recovered lost muscle volume. Furthermore, whilst isometric force per cm^2^ of mCSA and isokinetic force per kg of body mass were recovered to baseline values in both groups, not all elements of muscle function were fully restored in the older participants, in particular the ability generate force during the initial phase of muscle contraction (0–50 ms) remained impaired (Hvid et al. 2010). However, a more recent study by Hvid et al. (2014) suggests the time course of muscle strength recovery may require more scrutiny. Following 4 days of leg immobilisation, 7 days of recovery did not restore isometric or isokinetic knee extensor strength in older individuals, whilst younger individuals’ strength returned to baseline. The re-training regime used in the three studies mentioned implemented unilateral lower limb resistance exercise, and although it has been to shown to induce measurable gains in muscle size and strength in older individuals post hip replacement surgery (Suetta et al. 2004), the total training volume was not as great as that seen in the whole body resistance training in studies (Correa et al. 2013; Leenders et al. 2013). There is also no mention of control for dietary protein intake during the retraining periods in these studies, which may be an important factor in maximising the anabolic efficacy of resistance exercise in older individuals (Wall et al. 2014a). Moreover, consideration should be given to the fact that if recovery of muscle size was dramatically impaired in older individuals, then the long term consequence of disuse induced atrophy would likely result in a much more severe annual loss of lean tissue than the 0.5–1 % reported in the population data (Mitchell et al. 2012). It may be that older individuals need a longer or more intensive re-training period to recover from disuse induced atrophy compared to younger individuals, with the need for adequate protein consumption becoming crucial with advancing age (Greig et al. 2011; Hvid et al. 2014; Suetta et al. 2009). Nonetheless, it is clear that disuse induced atrophy poses a physiological challenge to older individuals; a concept illustrated in Fig. 1.

## The reduced activity model

Dramatic models of muscle unloading have allowed vital examination of the mechanisms resulting in muscle wasting, but these models are not necessarily an accurate reflection of the nature of unloading experienced by the majority of healthy, free-living individuals. Step-defined levels of activity have provided a useful index against which to correlate data on recognised markers of health (Tudor-Locke et al. 2013), and as such, a step-reduction model may be a feasible means of implementing controlled periods of reduced activity in experimental studies and an intervention comparable to real world scenarios of reduced activity. This step-reduction model has previously been implemented to explore the impact of limited ambulation on metabolic changes (Dixon et al. 2013; Knudsen et al. 2012; Olsen et al. 2008; Walhin et al. 2013). Reducing steps from ≈10,000 to ≈1500 per day for 2 weeks has been observed to significantly impair insulin sensitivity, attenuate postprandial lipid metabolism, and increase central adiposity (Thyfault and Krogh-Madsen 2011). These negative implications are widely considered to play major roles in the deterioration of metabolic health leading to chronic disorders such as Type 2 diabetes (Booth and Hargreaves 2011). However, a few of these studies have included measures of muscle mass changes from pre- to post-step-reduction which have provided the initial indication that measurable changes in leg muscle mass may be observed with as little as 2 weeks of reducing daily ambulation to less than 1500 steps (Table 1).

**Table 1:** Studies using step-reduction models with assessment of changes in muscle mass

Reference	Subjects	Step-reduction	Measures of muscle mass reported	Results
(Breen et al. 2013)	10 older adults; 5 females (72 ± 1 years)	5962 ± 695 to 1413 ± 110 steps/day for 14 days	DEXA; whole-body FFM, ALM, leg FFM, leg SMM, arm FFM, trunk FFM	↓ ALM, leg FFM, leg SM ≈ whole-body FFM, arm FFM, trunk FFM
(Knudsen et al. 2012)	9 young men (24 ± 3 years)	10,278 ± 715 to 1521 ± 131 steps/day for 14 days	DEXA; total FFM	≈total FFM[N.B. subjects overfed for a positive energy balance of 1978 ± 146 kcal/day]
(Krogh-Madsen et al. 2010)	10 young men (24 ± 2 years)	10,501 ± 808 to 1344 ± 33 steps/day for 14 days	DEXA; trunk LM, arm LM, leg LM	↓ leg LM ≈ trunk LM, arm LM
(Walhin et al. 2013)	26 young men; (25 ± 7 years)13 receiving 50 % surplus kcal daily, 12 receiving 50 % surplus kcal daily and undertaking 45 min running at 70 % VO2 max daily	12,562 ± 3520 to 3672 ± 866, and 10,544 ± 2756 to 3690 ± 400 steps/day for 7 days	DEXA; whole-body LM	↑ whole-body LM in both groups
(Devries et al. 2015)	30 older men; (70 ± 1 years)Undertaking 3 sessions/week of unilateral low-load resistance training (3 groups collapsed for muscle mass data)	7714 ± 809 to 1288 ± 62, and 7119 ± 797 to 1270 ± 88, and 6273 ± 981 to 1161 ± 107 steps/day for 14 days	DEXA; total FFM, ALM, leg FFM, leg SMM	↓ SR leg FFM↑ SR + RT leg FFM, SR + RT leg SMM≈ total FFM, SR leg SMM

*DEXA* dual energy X-ray absorptiometry, *FFM* fat free mass, *ALM* appendicular lean mass, *SMM* skeletal muscle mass, *LM* lean mass, *SR* step-reduction only limb, *SR* + *RT* step-reduction with resistance training limb

In young males (27.1 ± 5.7 years) who reduced activity from >10,500 to ≈1350 steps/day for 14-days Krogh-Madsen et al. (2010) observed a 2.8 % reduction in lean leg mass measured by DEXA. The authors described this finding as unexpected based on the view that muscle atrophy is likely to occur only in more dramatic forms of unloading. Further data from this study also revealed significant increases in mean intra-abdominal fat mass of the group from 693 to 740 mL (Olsen et al. 2008). To date, only one study has used this model to directly investigate muscle wasting and anabolic resistance in elderly individuals (Breen et al. 2013). As may have been expected based on previous data comparing older to younger participants in bed rest studies, Breen et al. (2013) found markedly more pronounced losses in muscle mass in 10 men and women aged 66–75 years walking <1500 steps/day for 14-days. The ≈76 % reduction in daily stepping in this study resulted in a 3.9 % loss of leg skeletal muscle, which is 25 % greater than the losses observed in younger individuals undertaking ≈88 % reduction in daily stepping (Krogh-Madsen et al. 2010). The difference in relative reduction of steps by the groups in these studies may be of note; the older participants had been taking <6000 steps/day before the intervention, less than half in comparison to the younger participants. It may be that lower levels of habitual physical activity in the older group predisposed those participants to greater loss of muscle tissue compared to the younger group. This may support the concept of habitually higher levels of physical activity eliciting a protective effect against anabolic resistance and therefore muscle wasting induced by short episodes of reduced activity. However, Krogh-Madsen et al. (2010) observed no correlation between the decline in number of steps from baseline and loss of lean leg mass between their younger participants. This leaves it unclear as to whether the greater muscle mass loss observed by Breen et al. (2013) was in fact a result of ageing per se rather than lower habitual physical activity prior to step reduction.

Breen et al. (2013) reported MPS response to provision of 25 g of egg protein to be reduced by ≈26 % following inactivity compared to pre-intervention, however there were no reductions in the associated signalling pathways (mTOR, p70^s6k^, and 4E-BP1) from pre- to post- step-reduction as seen in bed rest studies with older individuals (Drummond et al. 2012). Whilst this requires further examination, it may have been due to biopsy sample timing (4 h post-feeding); this was likely after peak phosphorylation which is generally considered to occur between one and 2 h post-feeding (Atherton et al. 2010). Alternatively, if anabolic signalling pathways were not down-regulated then other mechanisms could be explored, such as impaired amino acid transporter activity at skeletal muscle as observed due to bed rest in older adults by Drummond et al. (2012).

Another interesting contrast between the findings of Breen et al. (2013) and those of other step-reduction studies was the increase in plasma concentrations of TNF-α and CRP by ≈12 and ≈25 % respectively observed in the older group. These increases are modest, however Krogh-Madsen et al. (2010) saw no changes in plasma concentrations of TNF-α, IL-6 or IL-15 with 2-weeks of step-reduction to <1500 steps/day in young healthy males. In fact, Drummond et al. (2013) observed no change in serum cytokines, including TNF-α, across multiple time points of 7 days of bed rest in healthy older adults. Notably, participants of these studies had different body mass index scores; 24.8 kg/m^2^ in the Drummond et al. (2013) study compared to 29.0 ± 1.8 kg/m^2^ (accompanied by 31.9 ± 2.9 % total body fat) in the Breen et al. (2013) study. The significant increase in systemic inflammation may have been due to greater visceral fat leading to greater inactivity induced adipose derived cytokine release (Pedersen 2009). However, Dixon et al. (2013) also found no increase in markers of systemic inflammation in middle aged men reducing daily step count to <4000 steps for 7 days, despite one group being overweight (but active) and displaying a comparatively elevated CRP at baseline. These findings would suggest that markers of systemic inflammation in fact tend to remain stable when physical activity is reduced for short periods of time. Nonetheless, the role of systemic inflammation in progression of muscle wasting is of interest, and in an aged population with relatively high adiposity it may well have contributed to muscle atrophy and the decrease in MPS and as such requires further investigation (Breen et al. 2013).

An important consideration from Breen et al. (2013) was that no changes were elicited by 2 weeks of step reduction in isometric knee extensor torque. Previously, 10 days of bed rest in healthy older adults that resulted in 6.3 % losses in mean lean leg tissue brought about a concomitant drop in mean isokinetic knee extensor strength of 15.6 %, but ranging up to 23.1 % (Kortebein et al. 2007). As the methodology of strength measurement was essentially the same, it would be expected that accompanying muscle mass losses of nearly two-thirds of that observed by Kortebein et al. (2007) there would be at least some measurable loss of muscle strength due following the step reduction. Further investigation, possibly with different strength and functional measurement tools is warranted from this finding given that strength in older individuals commonly deteriorates at a greater rate than mCSA (Seene and Kaasik 2012). Nonetheless, it may have been that even the limited amount of ambulation permitted may have provided some protective effect against the loss of strength over the 2 week intervention.

The concept of re-introducing bouts of exercise whilst concomitantly reducing physical activity has been considered by Walhin et al. (2013) albeit with the primary aim of protecting against negative changes in insulin sensitivity and adipose tissue gene expression in the face of reduced activity and overfeeding in young healthy individuals. Twenty-six healthy young males (25 ± 7 years) randomised to two parallel groups took <1400 steps per day for 1 week alongside overfeeding by 50 % of habitual daily energy intake, with one of the groups also performing 45 min/day of running at 70 % of maximal oxygen uptake with additional overfeeding to maintain 50 % daily energy intake. Interestingly, increases in lean tissue were observed following 1 week of step count reduced <4000/day in both groups; 2.6 [1.9–3.4 (95 % confidence intervals)] kg in the group just receiving overfeeding with step reduction, and 1.0 (0.1–2.0) kg in the group additionally performing the daily exercise. The authors acknowledge that this is almost certainly due to muscle glycogen storage and accompanying water retention in muscle in response to overfeeding, which DEXA would non-discriminately identify as lean tissue. As a concept however, the re-introduction of daily exercise was effective in countering the effects of the reduced activity and energy surplus. More recently, Devries et al. (2015) explored the effects of introducing six bouts of low-load unilateral resistance exercise [three sets of leg press and leg extension to volitional failure at 30 % of one repetition maximum (1-RM)] during 14-days of step-reduction in older men and demonstrated remarkable protective effects against muscle atrophy compared to the non-exercised limb. This study undertook a three group design in which various combinations of daily nutritional supplements and test beverages for an infusion trial examining myofibrillar fractional synthetic rate (FSR) were provided to groups (5 g glycine per day, with 20 g whey protein isolate and 15 g glycine as test beverage; 5 g glycine per day, with 20 g micellar whey protein and 15 g glycine as test beverage; or 5 g citrulline per day, with 20 g micellar whey protein and 5 g citrulline as test beverage). Moreover, no differences were observed in muscle mass changes between groups, so this data was collapsed and demonstrated that whilst participants non-exercised leg lost 1.4 % skeletal muscle mass, the exercised leg in fact increased leg skeletal muscle mass by 1.4 % (Devries et al. 2015). The authors attribute the smaller loss of leg lean tissue observed in this study compared to that of Breen et al. (2013) to a potential cross-education effect of training the contralateral limb, although it is not clear whether the nutritional supplementation may have played a role in this attenuation of muscle atrophy. However, again step-reduction alone did not induce measureable loss of muscle strength, and more evidence of the potential cross-education effect of unilateral limb training comes from an increase in knee extension 1-RM in the untrained as well as trained limb (Devries et al. 2015). This study provides encouraging evidence that in real world scenarios of reduced activity periods, even relative.

## Possible mechanisms for exaggerated disuse induced muscle loss with ageing

Recent reviews by Bodine (2013) and Mallinson and Murton (2013) give detailed accounts of the current understanding of molecular mechanisms and signalling pathways involved in altering the balance of muscle protein turnover in response to disuse that results in muscle wasting. Though some of the primary mechanisms recognised in the literature will be highlighted in the current review, the emphasis here will be on drawing attention to how these mechanisms are altered by ageing induced changes in human physiology that may predispose older individuals to increased sensitivity to muscular disuse.

The total unloading models used in previous studies have allowed exploration of processes by which muscle protein turnover changes result in marked net muscle protein loss over time. To briefly summarise these findings, it has long been established that in humans the rate of MPS at rest falls rapidly at the onset of disuse and remains depressed until mechanical loading is reintroduced (Gibson et al. 1987). Thus far, fewer studies have addressed in vivo MPB rate responses to unloading due to the methodological complexities involved, particularly with regards to identifying the protein origin of tracer-labelled amino acids measured in arterial, venous, and intracellular sites. There is little direct evidence for elevation of MPB with unloading, however specific genes coding for skeletal muscle atrophy have been shown to be upregulated during bed rest. Expression of Muscle Ring Finger 1 (MuRF1) and Muscle Atrophy F-box (MAFbx) is hypothesised to regulate proteasome-mediated degradation of muscle proteins during atrophy, and temporal elevations have been reported under conditions of lower limb muscle unloading by Jones et al. (2004) and de Boer et al. (2007). A transient increase in MPB may be supported by data from Wall et al. (2014b) who reported marked elevations in mRNA expression of MAFBx and MuRF1 in a group undergo 5 days of limb immobilisation, compared with MuRF1 not being elevated after 14 days of immobilisation in a separate group. Generally, changes in MPB are not observed with unloading; for example Ferrando et al. (1996) did not observe any change in MPB rate across 14-days of bed rest, when measuring both whole body and skeletal muscle protein metabolism. However, Tesch et al. (2008) reported a transient increase in MPB lasting ≈72-h post onset of unloading, measured with a 3-methylhistidine microdialysis technique. The interpretation of data and methodology of this study have been respectfully criticised, in particular the choice of glucose as a marker of 3MeHis recovery and no direct measure of blood flow around the dialysis probe likely rendering this finding unreliable (Rennie et al. 2008). As in younger individuals, there is a lack of evidence for an associated increase in MPB playing a substantial role in disuse induced muscle wasting in older individuals (Drummond et al. 2012; Rennie et al. 2010). Overall, the dominant mechanism inducing atrophy during muscle disuse is still a matter of debate, as has been highlighted in recent discussions in the literature (Phillips and McGlory 2014; Reid et al. 2014).

During periods of normal physical activity, MPS is observed to increase markedly above basal levels following amino acid ingestion (Bohe et al. 2003; Symons et al. 2009). Reduced MPS at rest and in response to stimuli normally considered anabolic appears to be the driver of unloading induced muscle atrophy. Basal MPS accounted for almost half of the loss in muscle mass during 14-days of unilateral knee immobilisation in young men and women, and there was a blunted MPS response to amino acid infusion in the immobilised leg compared to the non-immobilised leg (Glover et al. 2008). It is thought that the combination of ‘anabolic resistance’ to protein feeding and the decreased basal MPS has a great enough influence on the balance of muscle protein turnover that any change in the rate of MPB does not play a substantial role in disuse induced muscle mass loss (de Boer et al. 2007; Gibson et al. 1987; Glover et al. 2008; Phillips et al. 2009).

The roles of mechanistic target of rapamycin complex 1 (mTOR) (Hall 2013) and p70 ribosomal s6 kinase (p70^s6k^) in up-regulation of protein translation initiation and protein synthesis in human skeletal muscle are well accepted (Rennie et al. 2004). Evidence from Cuthbertson et al. (2005) implicates suppressed expression and activation of these recognised anabolic signalling pathways in anabolic resistance in older individuals. Three hours after ingestion of 10 g of essential amino acids the phosphorylation of mTOR and p70^s6k^ only increased 2.7- and 3.5-fold respectively in older individuals, compared to 5.2- and 8.1-fold in younger individuals (Cuthbertson et al. 2005). Diminished anabolic signalling is further exaggerated by inactivity in older individuals (Drummond et al. 2012), despite immobilisation studies using young participants not demonstrating any decrease in mTOR pathway signalling (Bodine et al. 2001). Drummond et al. (2012) identified that after 7 days of bed rest the mTORC1 signalling pathway had a blunted response to EAA ingestion accompanied by significantly less MPS 3 h after EAA ingestion compared to pre-bed rest in older individuals. An earlier study showed that older individuals (70 ± 5 years) also exhibited anabolic blunting in response to resistance exercise; MPS expressed as total protein synthesised in the 4 h following unilateral leg extension exercise was ≈30 % lower compared to younger individuals (Kumar et al. 2009). Again, significantly lower phosphorylation of the downstream effectors of mTOR [p70^s6k^ and 4E binding protein 1 (4E-BP1)] were observed in the older compared to the younger group 1 h after exercise of an equal volume across a range of intensities above 60 % of one repetition maximum.

Symons et al. (2009) found no impairment in the mixed muscle FSR following provision of 113 g of lean beef (containing 30 g of protein) in older individuals compared to younger individuals. Furthermore, provision of 340 g of lean beef (90 g protein) had no further anabolic effect of mixed muscle FSR in either older or younger individuals. In later work from the same group, ingestion of the same 340 g lean beef meal post leg extension resistance exercise again showed no anabolic resistance in older compared to younger individuals (Symons et al. 2011). Rather than suggesting that anabolic resistance does not exist in older individuals, these studies highlight the importance of large doses of protein and resistance exercise to stimulate MPS. Moore et al. (2014) recently described the amount of protein relative to total body mass and lean body mass that saturates the dose response relationship of MPS rate in younger and older. Biphasic linear regression of a large data set of MPS rates to protein portion sizes from five studies undertaken at McMaster University identified 0.40 g/kg total body mass or 0.61 g/kg of lean body mass as sufficient to saturate MPS rate in older individuals. The studies mentioned from Symons et al. greatly exceeded the protein dose required to reach the MPS rate saturation; in Symons et al. (2009) the protein dose was 1.16 g/kg of total body mass, and in Symons et al. (2011) the dose was 1.80 g/kg of lean body mass. Thus, detection of anabolic resistance from studies providing very large doses of protein is less likely. Moreover, the protein dose relative to lean body mass required to achieve maximal myofibrillar FSR in older individuals is over twice that for younger individuals [0.25 ± 0.13 vs. 0.61 ± 0.28 g/kg lean body mass, p < 0.05 (Moore et al. 2014)].

An interesting observation of the data presented by Moore et al. (2014) is the relatively wide confidence intervals surrounding the dose required to achieve MPS rate saturation for the older individuals in comparison to the younger individuals. The authors note this large variation between older individuals, and suggest that this may be down to a combination of individual differences between participants, one of which may be habitual muscle contractile activity. Notwithstanding the strength of the studies used for analysis by Moore et al. (2014) habitual physical activity of the participants was not measured. Wall and van Loon (2013) have proposed that decreased physical activity status in elderly individuals may actually be the primary driver of anabolic resistance to protein feeding contributing to the overall negative flux in muscle protein turnover. Moreover, ageing itself is associated reduced appetite and decline in food intake, likely leading to lower daily dietary protein intake which would potentiate the problem of anabolic resistance to protein feeding (Visvanathan and Chapman 2010).

Another finding of note from Cuthbertson et al. (2005) was a four-fold difference in nuclear factor-κB (NF-κB) concentration in the elderly compared to young group. Activated by TNF-α and reactive oxygen species, NF-κB is a signalling protein for muscle atrophy and strongly linked with inflammation, however the mechanism by which NF-κB acts in ageing muscle is yet to be understood (Meng and Yu 2010). For example, Drummond et al. (2013) found no changes in either muscle NF-κB signalling or systemic inflammation during bed rest for 7 days in older adults despite 3.2 % loss of lean tissue, increases in toll-like receptor 4 (TLR4) signalling and expression of IL-6 mRNA in skeletal muscle. Interestingly, the authors observed a small increase in pro-inflammatory response within muscle tissue, and speculated that in light of the elevated TLR4 observed, short term extreme inactivity may induce an excessive pro-inflammatory response that may be ‘uncontrolled’. A review by Degens (2010) further describes the probable role of systemic inflammation, specifically circulating TNF-α, in exacerbating muscle loss in the elderly. Degens (2010) puts forward that evidence for the negative impact of TNF-α on satellite cell proliferation is suggestive of increasing the rate of muscle mass loss, albeit only once a certain ‘threshold’ of inflammation has been exceeded.

## Future directions

From the previous knowledge gained through implementing forms of complete lower limb unloading, and the more recent addition of data from step-reduction model studies, it is clear that more research is needed to define the relative contributions of levels of physical activity (both lifelong and habitual) and ageing per se to sarcopenia. Thereafter, further progress can be made in providing effective interventions to at least attenuate the progression of sarcopenia. Exploration of this more modest reduced activity intervention may be an important avenue for future research looking to inform on the impact of real world scenarios inducing muscle wasting in older individuals. Careful consideration should be given to the changes in appetite and dietary behaviour, particularly regarding daily protein consumption, in response to periods of step-reduction in older adults who may display anabolic resistance to feeding. On a wider scale, how such striking muscle wasting in a short space of time [≈4 % leg lean tissue loss in 2 weeks of step reduction (Breen et al. 2013)] impacts on the reported annual losses of 0.5–1 % in lean tissue observed at the population level is also of interest. If we are to assume that many older individuals do undergo periods of reduced activity as described, and experience muscle atrophy as a result, then it would appear that muscle mass accrual must be occurring in older individuals to restore deficits, or else the current population data would be suggestive of greater annual losses accordingly. This is in fact a positive notion in the context that this demonstrates the benefits of exercise in older individuals to restore lost lean tissue and potentially function. Moreover, it may be possible to develop understanding of how even a small dose of physical activity can be used to maintain muscle function during reduced activity periods, and subsequently regain lost muscle, or even provide a protective effect against muscle wasting before it occurs.
